# In Vivo Comparative Study on Acute and Sub-acute Biological Effects Induced by Ultrafine Particles of Different Anthropogenic Sources in BALB/c Mice

**DOI:** 10.3390/ijms20112805

**Published:** 2019-06-08

**Authors:** Francesca Farina, Elena Lonati, Chiara Milani, Luca Massimino, Elisa Ballarini, Elisabetta Donzelli, Luca Crippa, Paola Marmiroli, Laura Botto, Paola Antonia Corsetto, Giulio Sancini, Alessandra Bulbarelli, Paola Palestini

**Affiliations:** 1 School of Medicine and Surgery, University of Milano-Bicocca, 20900 Monza, Italy; francesca.farina@hotmail.it (F.F.); elena.lonati1@unimib.it (E.L.); chiara.milani@unimib.it (C.M.); elisa.ballarini@unimib.it (E.B.); elisabetta.donzelli@unimib.it (E.D.); luca.crippa@unimib.it (L.C.); paola.marmiroli@unimib.it (P.M.); laura.botto@unimib.it (L.B.); giulio.sancini@unimib.it (G.S.); alessandra.bulbarelli@unimib.it (A.B.); 2 POLARIS Research Centre, University of Milano-Bicocca, 20900 Monza, Italy; 3 Division of Neuroscience, San Raffaele scientific institute, 20121 Milan, Italy; admin@lucamassimino.com; 4 Department of Pharmacological and Biomolecular Sciences, University of Milano, 20100 Milano, Italy; paola.corsetto@unimi.it

**Keywords:** air pollution, ultrafine particles, inflammation, oxidative stress, diesel exhaust particles, biomass burning

## Abstract

Exposure to ultrafine particles (UFPs) leads to adverse effects on health caused by an unbalanced ratio between UFPs deposition and clearance efficacy. Since air pollution toxicity is first direct to cardiorespiratory system, we compared the acute and sub-acute effects of diesel exhaust particles (DEP) and biomass burning-derived particles (BB) on bronchoalveolar Lavage Fluid (BALf), lung and heart parenchyma. Markers of cytotoxicity, oxidative stress and inflammation were analysed in male BALB/c mice submitted to single and repeated intra-tracheal instillations of 50 μg UFPs. This in-vivo study showed the activation of inflammatory response (COX-2 and MPO) after exposure to UFPs, both in respiratory and cardiovascular systems. Exposure to DEP results also in pro- and anti-oxidant (HO-1, iNOS, Cyp1b1, Hsp70) protein levels increase, although, stress persist only in cardiac tissue under repeated instillations. Statistical correlations suggest that stress marker variation was probably due to soluble components and/or mediators translocation of from first deposition site. This mechanism, appears more important after repeated instillations, since inflammation and oxidative stress endure only in heart. In summary, chemical composition of UFPs influenced the activation of different responses mediated by their components or pro-inflammatory and pro-oxidative molecules, indicating DEP as the most damaging pollutant in the comparison.

## 1. Introduction

Air pollution is a global public health emergency that affects people of all ages in every part of the world. Nowadays, addressing ambient air pollution is the government priority, and the World Health Organization [1] issues air quality guidelines to defend the population in general and the most vulnerable in particular.

Numerous epidemiological studies have showed the effects of air pollution on the respiratory and cardiovascular systems. Short-term exposure to air pollution at higher levels reduces life expectancy by aggravating pre-existing respiratory and cardiovascular diseases [2]. Cardiovascular effects induced by particulate matter (PM) are linked to particle deposition in the lungs, to their translocation through the air-blood barrier to extra-pulmonary sites and the resulting systemic inflammation [3,4,5,6]. Particles’ deposition rates are strictly linked to the particle size: smaller particles have the highest deposition efficiency. Furthermore, ultrafine particles (UFPs, with an aerodynamic diameter less than 100 nm) are predominant when considering particle size number distribution while they contribute modestly when considering mass size distributions [7]. UFPs are probably the main mediators of PM toxicity due to the greater penetration efficacy into the respiratory system and to the higher translocation rates from the airways into the blood circulation [8,9]. Furthermore, UFPs are able to inhibit phagocytosis by enhancing their interaction with the alveolar epithelium [7]. Particles with a high surface area to mass ratio are able to adsorb potentially toxic organic chemicals or metals, increasing their capability to be a source of ROS [10,11]. Additionally, several studies evidenced that air pollutant composition is responsible for different toxic effects [12]: UFPs, due to their high organic chemicals content provide proportionally more redox cycling chemicals than larger particles [13]. Therefore, UFPs can show worse and different toxicity profiles in comparison to those of larger particles with the same composition, since their specific interaction with lung cells and their capability to translocate across the alveolar epithelial barrier [14]. Nonetheless, it cannot be excluded that systemic toxicity may be mediated also by PM or UFPs associated water-soluble components and/or biochemical mediators released in the lung and then translocated in blood circulation.

In Lombardy (North of Italy) diesel combustion and solid biomass burning are responsible for the 15% and the 50% of the primary fine particles production, respectively [15]. Combustion processes, if not complete, lead to the formation of particles of 15–30 nm in diameter, contributing largely to primary UFP emissions [16,17]. Diesel exhaust is a complex mixture of solid, condensed (or liquid), and gaseous corpuscular fractions [18]. The solid fraction is represented by diesel exhaust particles (DEP), with a bio-persistent core of about 10–30 nm in diameter [19]. These primary particles, composed of elemental carbon, can then agglomerate into larger soot aggregates with mean diameters of 60–100 nm [18]. The DEP surface can adsorb more than 300 chemical compounds, which include polycyclic aromatic hydrocarbons (PAHs), aliphatic hydrocarbons, quinones, transition metals and others [20]. Moreover, biomass burning (BB)-derived particles are obtained due to inefficient combustion that generates a multitude of partially oxidized organic chemicals, many of which have been associated with adverse health impacts [21].

In-vivo and in-vitro toxicological studies have evidenced diverse cardiovascular effects due to the particles’ origins and composition [22,23,24,25,26], which differently modulate inflammatory mediators and oxidative stress events [27]. Traffic-derived particles seemed to retain a higher inflammatory potential comparing to biomass particles in specific cellular models [28,29] but little is known about their systemic effects. Here we present a comparative in-vivo study aimed to elucidate the putative differences between the responses elicited by UFPs derived from diverse anthropogenic sources (DEP and BB). Intratracheal instillation of particulate matter in BALB/c mice is a useful and validated in-vivo model to study pollutants induced toxicity [30,31,32]. Therefore, we analyzed in this model how a short-term exposure or repeated exposures to DEP or BB could differently affect a panel of pro-inflammatory, cytotoxic, and oxidative stress markers in bronchoalveolar lavage fluid (BALf), lung and heart parenchyma, since they may not share the same biological mechanisms [33]. Thus, to clarify the specific cardiopulmonary damaging effects of short term and long-term exposure to different pollutants, could provide useful information for air quality standards guidelines and to clinical prevention for cardiovascular disease.

## 2. Results and Discussion

### 2.1. DEP Induced Higher Inflammatory Response than BB in BALf of Treated Mice

**Figure 1:**
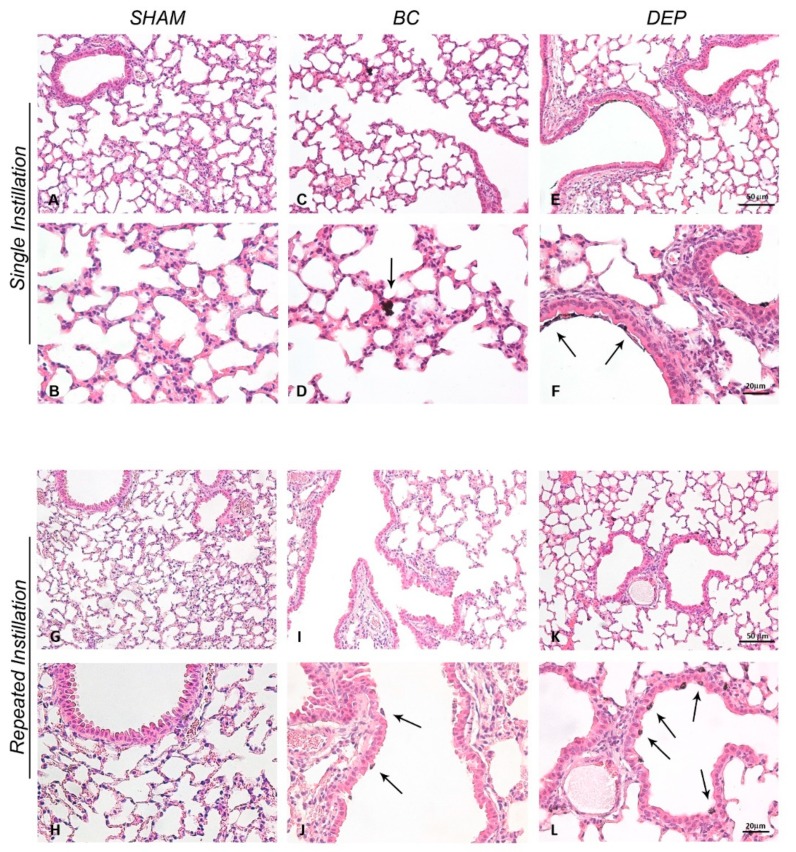
Lung histological analysis. Representative histological images of lung after single (**A**–**F**) or repeated (**G**–**L**) intratracheal instillation with 50 µg of BB (**C**,**D**,**I**,**J**) or DEP (**E**,**F**,**K**,**L**) in 100 µL 0.9% NaCl. Arrows indicate phagocytic cells containing particles in alveolar space and interstitium. (**A**,**C**,**E**; **G**,**I**,**K**) Bars = 50 μm; (**B**,**D**,**F**; **H**,**J**,**K**) Bars = 20 μm.

Cell-mediated immunity represents the first response triggered that indicates particles’ damage in pulmonary tissue. Under physiological conditions, healthy mice show a prevalence of alveolar macrophages (AMs) as resident immune cells in the lungs [34]. In response to exogenous agent inhalation, the recruitment of polymorphonuclear cells (PMNs) cooperate with AMs to phagocyte and eliminate particles [35]. Histological evaluation of lung parenchyma provided us qualitative information about the inflammatory status and potential pathological changes in the target organ. Phagocytic cells loaded with particles were observed in the bronchiolar epithelium either after acute and sub-acute exposure (Figure 1). In sham mice, lung parenchyma appeared normal (Figure 1A,B,G,H), while lung of UFPs-treated mice showed internalization of particles by phagocytic cells. Contrarily to BB-treated mice, where occasional particle internalization occurred (Figure 1C,D,I,J), DEP exposure showed several phagocytic cells containing particles in alveolar spaces and interstitium (Figure 1E,F,K,L).

In parallel, in the BALf of mice subjected to acute UFPs treatment, we observed a significant increase of PMN percentage in parallel to a decrease of AM percentage. Although the trend of cell count was similar after both UFPs treatment, DEP-exposure led to a significant increase of PMNs (71% of BALf total cells) and a significant decrease of AMs (29% of BALf total cells) (Table 1).

**Figure 2:**
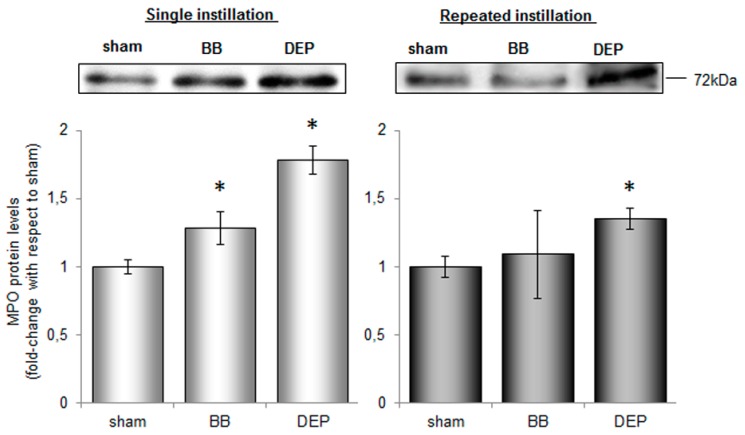
MPO protein levels in BALf after single and repeated instillations of BB and DEP. Representative immunoblotting images of MPO analysis in mice after single and repeated instillations with 50 µg of BB or DEP/100 µL 0.9% NaCl. MPO data are indicated as fold-change with respect to sham. Proteins have been normalized for corresponding total proteins revealed by Ponceau staining in each lane and the data are expressed as mean ± standard error (*n* = 6). Statistical differences were tested accordingly by One-way ANOVA followed by Bonferroni post-hoc comparison. * *p* < 0.05 vs. sham mice.

The high levels of lactate dehydrogenase (LDH), increasing about 1.5-fold after UFP treatment, led us to hypothesize the occurrence of necrotic damage (Table 1). The unvaried value of alkaline phosphatase (ALP) activity, a specific marker of Type I pneumocytes damage [36], suggests necrosis of AMs overloaded with particles in response to a failure of the macrophage clearance mechanism [37]. Indeed, LDH enzyme in BALf inversely correlated to AM percentage, pointing out that necrotic damage was probably directly affecting immune cells resident in the lung. The influx of PMNs (i.e., neutrophils 99%) is strongly associated with an acute inflammatory reaction [38]. Neutrophils contain four different classes of granules: azurophilic, specific, gelatinase granules and secretory vesicles. Neutrophils degranulation, occurring after their stimulation, induces a release of inflammatory markers into the extracellular space: specifically myeloperoxidase (MPO) is released from azurophilic granules while matrix metalloproteinase-9 (MMP9) from gelatinase granules [39]. Both these proteins when released are involved in lung injury [39,40], modulating the release of other biochemical mediators and extracellular matrix remodelling [41]. Accordingly, short-term exposure to UFPs leads to significant increase in MPO and an increasing trend in MMP9, indicating activation of infiltrated neutrophils in both the treatments. Interestingly, DEP promoted a stronger increment of MPO (2-fold) (Figure 2) compared to sham (3-fold compared to BB) and a 10-fold increase in MMP9 amount (Table 1), in line with the major recruitment of neutrophils observed. Moreover, BALf total protein concentration doubled after DEP treatment, suggesting an increased permeability of the air-blood barrier (Table 1). Indeed, the acute inflammatory event is associated with vascular leakage, essential to allow leucocyte migration to inflammation site [42,43].

**Figure 3:**
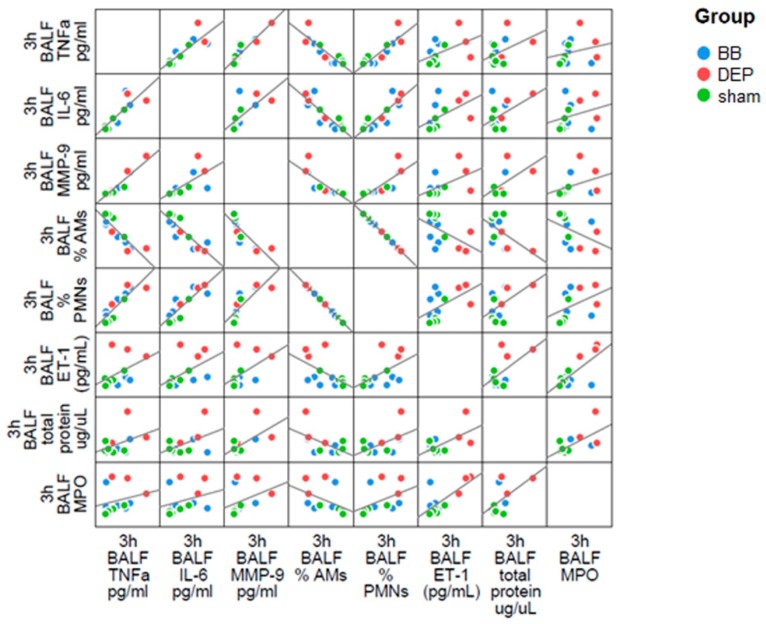
Correlation analysis in BALf. Dispersion plot showing the co-distribution of BALf parameters in acute treatment. Linear regression correlations are displayed as black lines.

The higher toxic potential of DEP is also revealed by the increased ET-1 concentration in the BALf, probably secreted from lung endothelial cells, as well as by AMs. ET-1 could promote acute lung injury, inducing cytokine production and activating neutrophils to release oxygen radicals [44]. Actually, levels of TNF-α and IL-6 pro-inflammatory cytokines were 4-fold augmented in BALf of DEP-treated mice. Although cytokine increment is not significant, TNF-α and IL-6 had a positive correlation between themselves as well as with the influx of PMN percentage in the BALf and MMP9 amount (Figure 3).

Indeed, IL-6 could dictate the profile of leukocyte recruitment during the inflammatory response [45] while TNF-α may stimulate specific degranulation and MMP9 release [39]. Similarly, MPO positive correlation with PMNs percentage and ET-1 could depend by active neutrophils stimulated to release MPO. Our evidences pointed out UFP ability to activate in the lung a local inflammatory response during the acute insult, and showed higher effects induced by UFP derived from diesel combustion compared to the ones resulting from biomass burning.

With the sub-acute treatment, we did not observe a significant change of BALf parameters among the three groups, suggesting some kind of adaptation to the inflammatory status caused by desensitisation after repeated exposures. After DEP-treatment, MPO protein level resulted the only parameter significantly higher than sham, although it appeared lower when compared with the acute exposure (Figure 2). We supposed that this event was due to a lower recruitment of PMNs after repeated instillations with respect to the single one, according to a global re-establishment of others BALf parameters. Finally, high MPO protein levels might be associated to the persistence of particle-loaded phagocytic cells in lung parenchyma after DEP exposure. MPO, hence, results a useful biomarker for evaluating the pulmonary toxicity of inhaled chemicals since high concentrations of protein keep on for a long time after instillation [46].

### 2.2. Both UFPs Induced Inflammatory Response in Lung and Heart Parenchyma of Treated Mice

The heart is the first organ directly reached by the bloodstream coming from the lungs, through the pulmonary circulation. Thus, although histologic analyses did not show infiltration of phagocytic cells overloaded with BB and DEP particles in the hearts of treated-mice (data not shown), UFP components or pro-inflammatory molecules produced in lung might immediately translocate and reach the cardiac tissue.

**Figure 4:**
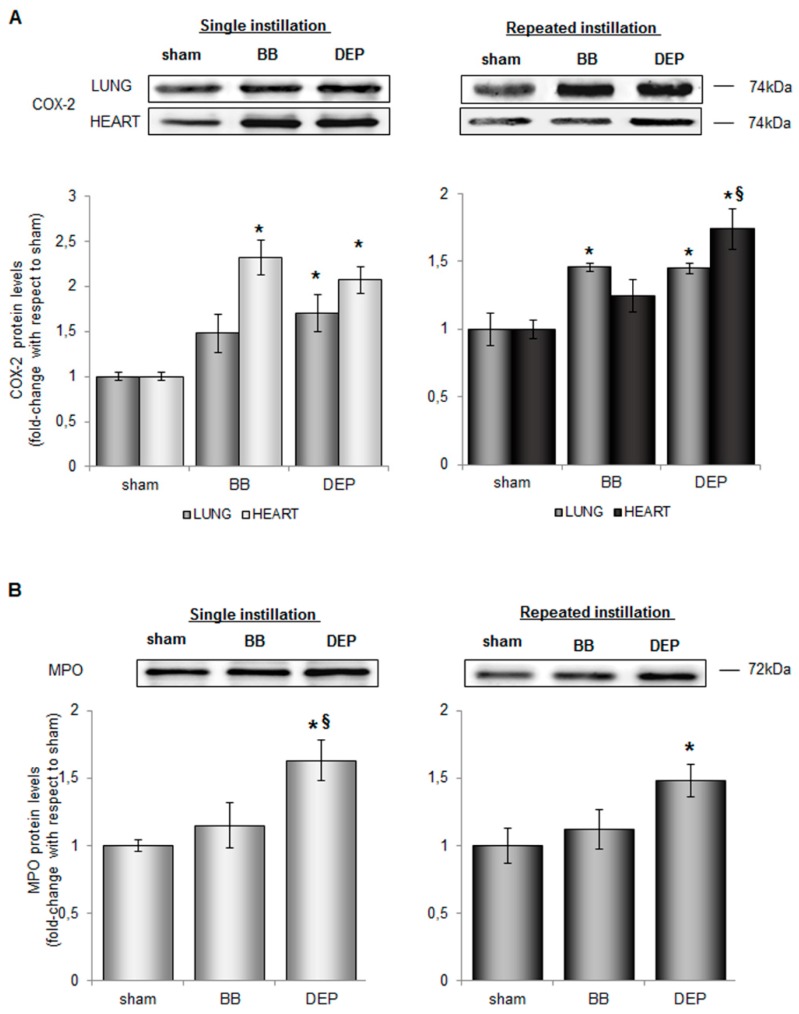
Inflammatory markers protein levels in lung and heart parenchyma. Representative immunoblotting images of COX2 analysis in mice lung and heart (**A**) and of MPO in heart parenchyma (**B**) after single and repeated instillations with 50 µg of BB or DEP/100 µL 0.9% NaCl. Histograms display COX2 and MPO as fold-change with respect to sham. Proteins have been normalized for corresponding total proteins revealed by Ponceau staining in each lane and the data are expressed as mean ± standard error (*n* = 6). Statistical differences were tested accordingly by One-way ANOVA followed by Bonferroni post-hoc comparison. * *p* < 0.05 vs. sham mice; § *p* < 0.05 vs. BB.

Airway diseases have been already associated with cyclooxygenase 2 (COX-2) regulation at both gene and protein levels in-vitro and in-vivo following DEP treatment [47,48,49], but little is known about BB. In order to understand if the acute inflammation observed was associated to this key modulator of airway inflammation [50], COX-2 protein levels were evaluated in lung and heart parenchyma. In acute treatment, instillation of both BB and DEP caused an increase of COX-2 protein levels with a stronger increase in the heart (about 2-fold) than in lung (about 1.7-fold) with respect to sham (Figure 4A). Interestingly, in sub-acute treatment, COX-2 levels were maintained higher 24 h after the last intratracheal BB or DEP instillation (Figure 4A). Considering that in the cardiovascular system, COX-2 activity is associated with the control of leukocyte-endothelial cell adhesion and platelet aggregation [51] its activation may potentially be involved in endothelial dysfunction and, in turn, in atherosclerosis progression [52]. Accordingly, MPO levels significant increased respect to sham after both acute and sub-acute DEP-treatment (about 1.7-fold and 1.5-fold respectively) (Figure 4B), also in the heart parenchyma, while BB determined not significant increases. This data results very remarkable considering that the MPO locally released by immune cells could transcytoses to the sub-endothelial matrix from vascular endothelium [53].

Thus, elevated MPO levels after DEP treatment might result in its higher passage in heart parenchyma with an increasing cardiovascular risk [54] although the histochemical data here reported did not completely supported this hypothesis. In UFP-treated mice, lung injury activated MMP9 protein through neutrophil degranulation, as an effect of local inflammation [39]. Thus, considering the observed MMP9 increment in BALf, a possible metalloproteinase activation following BB or DEP intratracheal administration was explored in living mice, using the FMT 1500 fluorescence imaging system and the MMPSense^TM^ 750 FAST. This probe is a matrix metalloproteinase (MMP) agent that is optically silent upon injection and produces a fluorescent signal after cleavage by inflammation related MMPs (MMP-2, 3, 7, 9, 12, and 13). Since the recommended optimal imaging time point is 6–24 h post-injection of the probe, the MMP activity evaluation after 3 h from single instillation was not possible. For this reason, in order to compare the effects of the single and the repeated exposures, in-vivo imaging was performed 24 h post-injection from the first (day 1) and the last (day 6) instillation in the sub-acute treatment.

**Figure 5:**
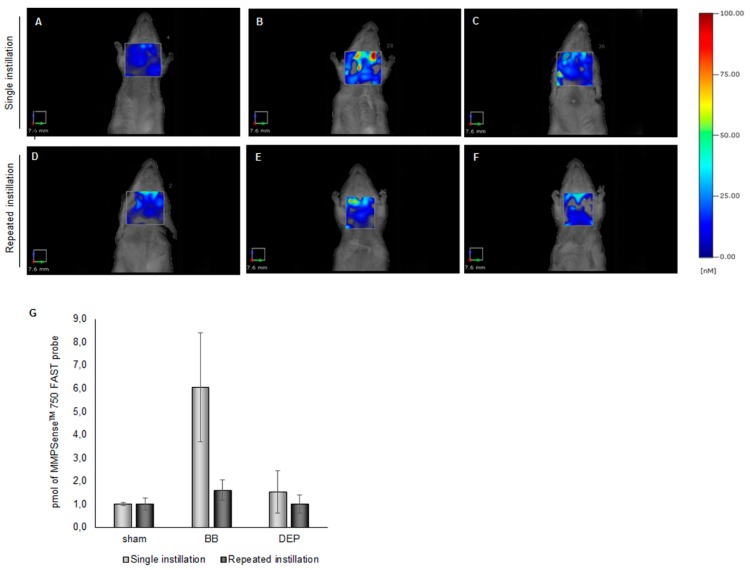
FMT of mouse thoracic region after the first (**A**–**C**) and the last (**D**–**F**) instillation of BB and DEP. Representative FMT images of sham (**A**), BB- (**B**) and DEP- (**C**) treated mice brain obtained 24 h after the first intratracheal instillation with 50 µg of BB or DEP/100 µL 0.9% NaCl. Representative FMT images of sham (**D**), BB- (**E**) and DEP- (**F**) treated mice brain obtained 24 h after the last intratracheal instillation with 50 µg of BB or DEP/100 µL 0.9% NaCl. Each figure represents the status evidenced examining two mice for every treatment, and histograms (**G**) are representative of pmol of MMPsense 750 FAST probe. Data are expressed as mean ± standard error.

In the thoracic region, 24 h after the first instillation, the MMPSense^TM^ 750 FAST showed a 6-fold increase for BB (Figure 5B) and a 1.5-fold increase for DEP (Figure 5C), comparing to sham (Figure 5A). Similarly, 24 h after the last instillation, the signals for BB were found to be higher (1.6-fold) (Figure 5E) with respect to sham as well as to DEP (Figure 5F) that returned comparable to control levels (Figure 5D). Interestingly, initial data have indicated that BB treatment was more effective than DEP in MMPs activation (Figure 5G) although the biochemical and histological analysis here reported showed a higher impact of DEP.It is possible to speculate that since BB is enriched in Mn, an inductor of MMP proteins [55], specific metal and trace elements concentration may have a primary role in the activation of different pathways along the time. Indeed BALf analyses were performed after 3 h from the single instillation, while FMT, for techincal esigence, after 24 h.

Moreover, MMP9 protein level increment in BALf after DEP (Table 1) was probably due to a release from neutrophils, while FMT give us a picture of the all MMPs activation in the whole thoracic area, evaluating not only BALf but all the organs involved in the cardiovascualar injury.

### 2.3. DEP Induced Higher Oxidative Stress Response than BB in Lung and Heart Parenchyma of Treated Mice

Notably, DEP exposure had the major stress outcome, while no significant changes were observed after BB exposure. Longhin et al. [56] using the same UFP batches in in-vitro models, reported that DEP exposure induced a stronger modulation of gene transcription as compared to BB in term of both differentially expressed genes, temporal patterns and pathway activation. The authors showed that DEP was enriched in polycyclic aromatic hydrocarbons (PAHs), such as pyrene, phenanthrene, benzo[a]anthracene and dibenzo[a,h]anthracene which may induce oxidative stress.

**Figure 6:**
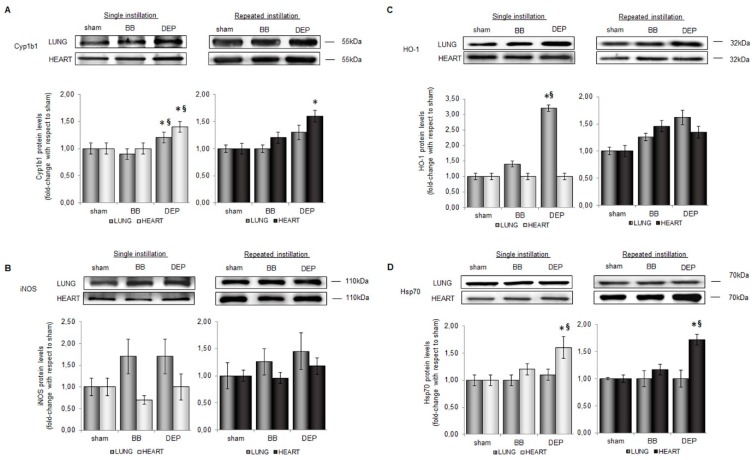
Oxidative stress markers protein levels in lung and heart parenchyma. Representative immunoblotting images of Cyp1b1 (**A**), iNOS (**B**), HO-1 (**C**), Hsp70 (**D**), in lung and heart parenchyma after single and repeated instillations with 50 µg of BB or DEP/100 µL 0.9% NaCl. Histograms display Cyp1b1, iNOS, HO-1 and Hsp70 as fold-change with respect to sham. Proteins have been normalized for corresponding total proteins revealed by Ponceau staining in each lane and the data are expressed as mean ± s.e.standard error (*n* = 6). Statistical differences were tested accordingly by One-way ANOVA followed by Bonferroni post-hoc comparison. * *p* < 0.05 vs. sham mice; § *p* < 0.05 vs. BB.

Indeed, PAHs are inducers and substrates of cytochrome P450 enzymes like Cyp1b1 [57], an enzyme implicated in their detoxification. Transformation of PAHs by cytochrome P450 could lead to reactive oxygenated intermediates able to interact with and oxidize cellular macromolecules [58,59]. Cyp1b1 increased significantly immediately after the DEP single instillation in lung and heart parenchyma, whereas during sub-acute treatment significant levels of this enzyme occurred only in cardiac tissue (Figure 6A).

Differently, analysis of inducible nitric oxide synthase (iNOS), a pro-oxidant protein involved in NO production [60], showed an increasing trend only in the lung but not in heart parenchyma after UFPs exposure (Figure 6B).

In parallel to the activation of pro-oxidant proteins, in DEP-treated mice we observed the up-regulation of antioxidant and protective enzymes, such as heme oxygenase 1 (HO-1) and heat shock protein 70 (Hsp70) (Figure 6C,D). HO-1 activity plays an important role in the antioxidant response since it generates anti-inflammatory and anti-oxidant molecules in the rate-limiting step of heme degradation [61]. After the acute exposure to DEP, HO-1 significantly increased (3-fold) with respect to sham (Figure 6C), suggesting an immediate reaction of lung tissue to counteract the oxidative stimulus, as already demonstrated in response to particulate matter [6,30,62]. The antioxidant enzyme activation persisted also after the last instillation of the sub-acute treatment with DEP although dampened with respect to a single event. Interestingly, while HO-1 increased in lung parenchyma and Hsp70 levels did not, the opposite was observed in the cardiac tissue. Hsp70 acts as a chaperone in maintaining normal cell function after different insults, including oxidative stress and pro-inflammatory cytokines [63]. In cardiac tissue protein levels increased immediately after acute exposure (Figure 6D) possibly in relation to the high content of PAHs, Zn and V, adsorbed onto DEP. These compounds were in fact described as inducers of Hsp70 expression [64,65]. As observed for Cyp1b1, Hsp70 levels were still higher after sub-acute exposure to DEP. We speculate that, since PAHs are able to form PAHs-albumin adducts, PM-desorbed PAHs in the lung and bounded to albumin as a carrier could be able to pass the alveolar epithelium and endothelium and eventually reach other target tissues [66,67], suggesting a delayed translocation of PAHs rather than of UFPs. Interestingly recently it has been shown in cells exposed to real world UFPs the high correlation of HO-1, Cyp and inflammatory proteins to specific chemical compounds associated to vehicular traffic and biomass burning emissions [68] again suggesting that the release of such compounds from soot particles may be relevant to understand particles-associated health effects.

Moreover, MPO high levels in heart of DEP-treated mice could be another potential source of oxidative stress, since ROS produced by its enzymatic activity may affect the PAHs transformation to highly reactive intermediates [32,69].

The decrease of pro- and anti-oxidant protein levels in lung under sub-acute DEP treatment, compared to acute event, suggests that the antioxidant systems were active only until the pro-oxidant stimuli were high in the first deposition site. Then, the antioxidant machinery tended to burned out, although it might rest sensitive to successive acute events. On the other hand, frequent exposure to DEP might sustain prolonged oxidant stress environment in close or distant organs from pollution instillation locus.

### 2.4. The Interdependence between Oxidative Stress and Inflammation Following DEP-Exposure

**Figure 7:**
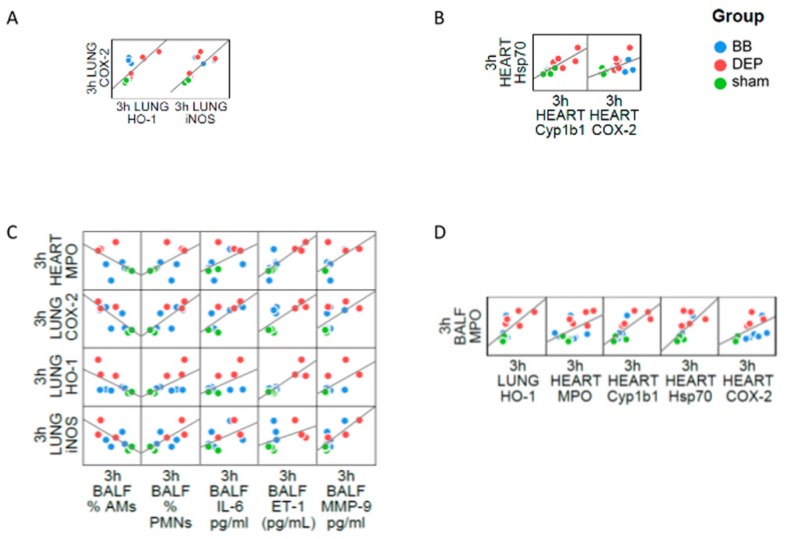
Correlation analysis between inflammation and oxidative stress in acute treatment. Dispersion plot showing the co-distribution of markers intra-lung (**A**), intra-heart (**B**), inter-lung/heart and BALf (**C**,**D**). Linear regression correlations are displayed as black lines.

Oxidative stress and inflammation are pathophysiological events strictly connected, cause and consequence of each other in several diseases [70,71], including those induced by air pollution exposure. Here we showed the co-presence of both processes, and their interplay among the organs evaluated. At intra-organ level the inflammatory marker COX-2 increase was associated with both HO-1 and iNOS protein levels after the single instillation in the lung (Figure 7A), as well as the inflammatory marker correlated with Hsp70 protein in heart parenchyma (Figure 7B).

Moreover, during the inflammatory processes activated neutrophils and macrophages produces large amounts of ROS that might diffuse out of the phagocytic cells and induce localized oxidative stress and tissue injury [72]. Indeed, inflammatory and oxidative stress markers expressed in different fluids or tissues of DEP-treated mice were statistically correlated (Table S1). A condition of systemic inflammation was demonstrated by the correlations among lung COX-2 and heart MPO with PMNs, AMs, IL-6, MMP9 and ET-1 concentrations in BALf. In this context, it is worth to note that lung HO-1 and iNOS were positively associated to the above mentioned pro-inflammatory BALf parameters (Figure 7C). This evidence indicated a strong association between anti/pro-oxidant proteins production and acute inflammation, once more confirmed by the correlation between MPO concentration in BALf and expression of inflammatory (MPO, COX-2) and oxidative stress (Cyp1b1, Hsp70) markers in heart, as well to HO-1 in the lung (Figure 7D).

**Figure 8:**
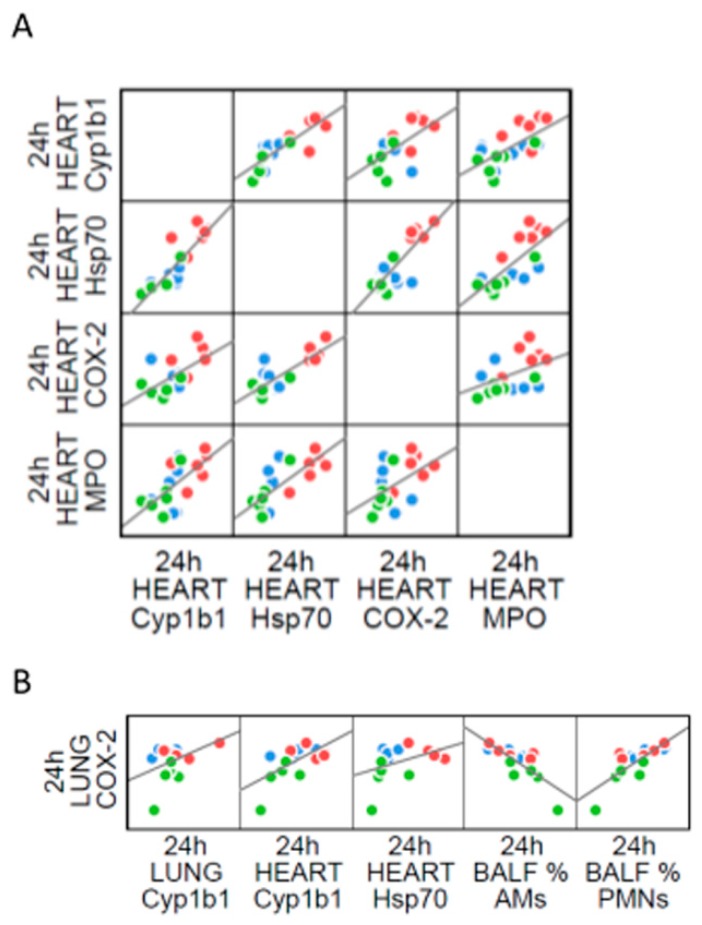
Correlation analysis between inflammation and oxidative stress in sub-acute treatment. Dispersion plot showing the co-distribution of markers intra-heart (**A**) and of lung COX2 with heart and BALf parameters (**B**). Linear regression correlations are displayed as black lines.

Interestingly, after sub-acute treatment we observed a correlation among all the markers analyzed in heart parenchyma (Figure 8A). In parallel, COX-2 protein levels in lung correlates with the phagocytic cells influx in BALf and to Cyp1b1 and Hsp70 in heart (Figure 8B). This evidence reinforcing the hypothesis that under repeated exposures to UFPs, pro-inflammatory and oxidative molecules produced by initial deposition in the lung, reach further organs through circulation. All correlations analyses are reported in Table S2.

## 3. Materials and Methods

### 3.1. UFPs Characterization

DEP and BB batches were provided by ENEA (Agenzia Nazionale per Le Nuove Tecnologie, L’Energia e Lo Sviluppo Economico Sostenibile) in the framework of the project “Biological effects and human health impacts of ultrafine particles sources” lead by Prof Camatini of the POLARIS research centre. Particle sampling procedures and their characterization are extensively reported elsewhere [56]. Briefly, DEP were sampled from five Teflon filters (Whatman pure Teflon filters, Maidstone, United Kingdom) obtained by a diesel Euro 4 light-duty vehicle without an anti-particulate filter fuelled by commercial diesel and run over a chassis dyno, while BB particles were collected from five Teflon filters (Whatman pure Teflon filters) obtained by a modern automatic 25 kW boiler propelled by a prime quality spruce pellet. Transmission electron microscopy (TEM) and scanning electron microscopy (SEM) images of both diesel and biomass samples showed aggregates of round carbonaceous particles lower than 50 nm; in addition, biomass samples showed the presence of ash particles that completely dissolved in aqueous media [56]. PAHs and transition metals (Fe, Zn, Cr, Pb, V and Ni) concentration was higher in DEP compared to BB, which conversely resulted enriched in elements typical of wood combustion, such as Mn, K and S. Below we provide a table (Table 2) summarizing the results obtained by Longhin and colleagues [56].

### 3.2. Animals and Treatments

#### 3.2.1. Animal Housing

Male BALB/cOlaHsd mice (7–8 weeks old, 20–25 g weight) were purchased from Envigo (San Pietro al Natisone, Italy) with a health monitoring report based on FELASA recommendations. Mice were housed in plastic cages in groups of three for five days to acclimate to the housing facility. They were housed under controlled environmental conditions (temperature 19–21 °C, humidity 40–70%, lights on 7 a.m.–7 p.m.) with food and water administered ad libitum. Animal use and care procedures were approved by the Institutional Animal Care and Use Committee of the University of Milano-Bicocca (protocol 02-2014) and complied with guidelines set by the Italian Ministry of Health (DL 26/2014 “Application of the Directive n. 2010/63/EU on the protection of animals used for scientific purposes”).

#### 3.2.2. Intratracheal Instillation

Animal testing has been carried out in the morning in the housing facility. Animals were randomly divided into three experimental groups (three mice for each group) for exposition to the different UFPs both in acute and sub-acute treatment: sham (isotonic solution); BB-treated mice and DEP-treated mice. The experiments were replicated twice, for a total of 6 shams, 6 BB- and 6 DEP-treated mice for each type of treatment. The sample size is in line with previously published data on air pollutant adverse effects [30,32] with the aim to minimize the number of animals employed. Every mouse, numbered from 1 to 9 (1–3 CTRL; 4–6 DEP; 7–9 BB), was singularly exposed to a mixture of 2.5% isoflurane (flurane) anesthetic gas and kept under anesthesia during the whole instillation procedure. Once a deep stage of anesthesia was reached, mice were intratracheally instilled by means of a MicroSprayer Aerosolizer system (MicroSprayer Aerosolizer- Model IA-1C and FMJ-250 High-Pressure Syringe, Penn Century, Philadelphia, PA, USA), with 100 µL of isotonic saline solution (sham) or 50 µg of BB or DEP in 100 µL of isotonic saline solution (exposed). The entire procedure of intratracheal instillation is fully described in previous works [62,73]. UFP dosage was chosen considering previous in-vivo investigation [74,75,76] that suggested 50 μg/mice dose as the most appropriate one for detection of acute and sub-acute inflammatory changes in lung exposition. Although, UFPs is at ambient background levels < 2 μg/m^3^, it can increase severalfold at locations with high volumes of traffic or during high-pollution episodes [77]. UFP dose here used is the lowest dose to establish lung inflammatory response in exposed mice.

**Figure 9:**
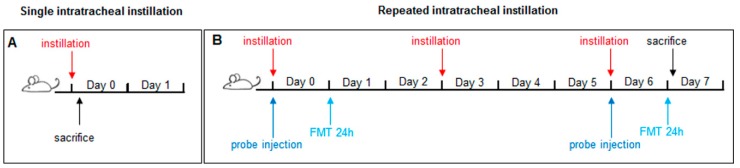
Schematic representation of BALB/c acute (**A**) and sub-acute (**B**) treatment and FMT.

According to our published data with PM [62], 3 h after instillation is the proper time point to evaluate some protective or inflammatory markers most likely expressed in acute phase (Figure 9A).

Instead, mice subjected to three repeated instillations of 50 μg UFPs/instillation every three days are consider representative of a sub-acute exposure (Figure 9B) [30,31,32,78]. Three h after a single instillation or 24 h after the last instillation of the repeated exposure protocol, mice of each experimental group (sham, BB and DEP-treated) were anesthetised by gas to minimize suffering and euthanized with cervical dislocation. The Broncho Alveolar Lavage Fluid (BALf), lung, heart of every mouse have been collected as described previously [30,32] and analyzed for markers of cytotoxicity, inflammation and oxidative stress.

### 3.3. Bronchoalveolar Lavage Fluid Analysis

The BALf procedure, pellets, and supernatant recovery were performed as described previously [62,73]. Briefly, the trachea was exposed, cannulated and secured with suture thread, and three in-and-out washes with 0.6 mL of isotonic saline solution were performed [47]. The efficacy of BALf collection ranged from 50% to 90% of the total solution injected. The BALf was centrifuged at 1500× *g* for 15 min at 4 °C [79] and pellets collected for cell counts. Supernatants were divided into aliquots and appropriately stored for subsequent biochemical analyses.

#### 3.3.1. Cell Counts

Total and differential cell counts have been performed according to the literature [62,73]. Briefly, BALf pellets were resuspended in 500 μL of DMEM (10% FBS, 1% penicillin-streptomycin, 1% glutamine), and total cell counts performed with a Burker chamber, using the Trypan Blue exclusion method. A cell aliquot (240,000 cells, 800 cells/μL) was smeared in duplicate onto slides using StatSpin Cytofuge 2 (Beckman Coulter Brea, CA, USA) 40× *g* for 7 min at room temperature. Subsequently, the smears were stained with Diff Quik (Medion Diagnostic, Miami, FL, USA) for cell differential count, according to the manufacturer’s instructions. Macrophages, polymorphonuclear leukocytes (PMNs) and lymphocytes were identified by their characteristic shapes.

#### 3.3.2. Biochemical Analyses on cell-free BALf Supernatants

Pro-inflammatory cytokines, such as tumor necrosis factor alpha (TNF-α) and interleukin-6 (IL-6), as well as the metalloproteinase MMP9, were analyzed by means of Luminex Screening Assay by LABOSPACE (Milano, Italy). The commercially available kits for alkaline phosphatase (ALP) activity (DALP-250 QuantiChrom Alkaline Phosphatase Assay Kit, Gentaur Molecular, (San Jose, CA, USA), lactate dehydrogenase (LDH) activity (DLDH-100 QuantiChrom Lactate Dehydrogenase Kit, Gentaur Molecular) were employed according to the manufacturers’ instructions.

Endothelin-1 (ET-1) and heat shock protein 70 (Hsp70) concentration were assayed by means of commercially available ELISA kits (Endothelin-1 ELISA kit and HSP70 high sensitivity ELISA kit, Enzo Life Sciences, (Rome, Italy), according to manufacturer’s instructions.

Total Antioxidant Capacity (TAC) was evaluated by means of TAC Colorimetric Assay Kit (BioVision, Milpitas, CA, USA) according to manufacturer’s instructions, in order to analyse the overall cell capability to counteract reactive oxygen species (ROS). Briefly, Cu^2+^ ion is converted to reduced Cu+ ion by both antioxidant small molecule and protein. The reduced Cu+ is chelated with a colorimetric probe giving a broad absorbance peak around 570 nm, proportional to the total antioxidant capacity. All measurements performed were normalized to the total protein content evaluated by means of the bicinchoninic acid assay (BCA). Myeloperoxidase (MPO) analysis was performed by western blotting, as described in Section 3.6.

### 3.4. Lung and Heart Homogenization

Lung and heart homogenates of sham, BB- and DEP-treated mice have been prepared and proteins were processed as previously described [30]. Briefly, the lungs and the heart of sham and treated mice, at the end of BAL procedure, were quickly excised from the chest and washed in ice-cold isotonic saline solution. The left lobes were dissected and submitted to histology, the right lobes were preserved for the western blot analyses. For protein assays, lungs were minced at 4 °C, suspended in NaCl 0.9%, briefly homogenized for 30 s at 11,000 rpm with Ultra-Turrax T25 basic (IKA WERKE, Staufen, Germany) and sonicated for other 30 s. Protein amount was determined by BCA method (Sigma Aldrich, St Louis, MO, USA).

### 3.5. Histopathological Analysis

Once excised, lungs and hearts from sham BB- and DEP-treated mice were washed with 0.1 M phosphate buffered saline (PBS, pH 7.4), fixed by immersion in 10% buffered formalin o/n at RT, embedded in paraffin (Embedding Center Leica EG1160, Leica Biosystem, Wetzlar, Germany), cross-sectioned at 3 µm thickness by a rotary microtome, mounted on slides, and stained by Haematoxylin and Eosin (HE). Finally, they were observed under a light microscope (Nikon Eclipse 50i, Melville, NY, USA). Representative images were captured with a digital camera (Nikon Digital Sight DS-2Mv). Data were obtained from two sham, two BB- treated and two DEP-treated mice.

### 3.6. Electrophoresis and Immunoblotting

Protein analysis was performed by SDS-PAGE electrophoresis/western blot, loading 30 μg each sample. Proteins were transferred to a nitrocellulose membrane (Amersham, GE Healthcare Europe GmbH, Milano, Italy), and revealed by immunoblotting with specific antibodies: rabbit polyclonal heme oxygenase-1 (HO-1) (1:200)(sc-10789; Santa Cruz Biotechnology™, Dallas, TX, USA), rabbit polyclonal inducible nitric oxide synthase (iNOS) (1:200) (sc-8310; Santa Cruz Biotechnology™), rabbit polyclonal cytochrome 1b1 (Cyp1b1) (1:200) (sc-32882; Santa Cruz Biotechnology™), goat polyclonal heat shock protein 70 (Hsp70) (1:200)(sc-10-70; Santa Cruz Biotechnology™), rabbit polyclonal cyclooxygenase 2 (COX2) (1:1000)(#4842; Cell Signalling Technology^®^, Danvers, MA, USA), and rabbit polyclonal myeloperoxidase (MPO) (1:200) (sc-16128-R; Santa Cruz Biotechnology™). The secondary antibodies were appropriate horseradish peroxidase (HRP)-conjugated goat anti-rabbit (1:5000) (31460 Thermofisher Scientific™ Waltham, MA, USA) or donkey anti-goat (1:2000) (sc-2020; Santa Cruz Biotechnology™). Immunoblot bands have been analyzed and the optical density quantified by LAS4000 (GE Healthcare, Marlborough, MA, USA); all the data have been normalized to Ponceau staining (Sigma Chemical Co., Milano, Italy) [78].

### 3.7. Fluorescence Molecular Tomography

Fluorescence molecular tomography (FMT^®^) is an imaging technique for improved fluorescence signals localization and quantification in deep tissue. After hair removal, male BALB/c mice were injected intravenously with MMPSense^TM^ 750 FAST probe, immediately before the first and the last instillation of the sub-acute treatment. MMPSense^TM^ 750 FAST probe is a matrix metalloproteinase (MMP) activatable agent that is optically silent upon injection but after cleavage by MMPs becomes fluorescent. The recommended optimal imaging time point is 6–24 h post-injection of the probe, to allow its distribution and the decrease of background signal; thus the evaluation after 3 h from single instillation was not possible. For this reason in-vivo imaging was performed during sub-acute treatment after 24 h from the first (day 1) and the last (day 6) instillation, in order to compare the effects of the single and the repeated exposures., Analyses was performed using the FMT 1500TM In Vivo Imaging System (PerkinElmer, Waltham, MA, USA).

The total amount of fluorophore in a selected three-dimensional region of interest (ROI) was calculated by the TrueQuant software. ROI was drawn with the same size each sample and so as to include the whole cardiopulmonary area. ROIs drawing was executed in a blind manner by an operator unaware of the experimental origin of the specimens in order to eliminate any operator bias.

### 3.8. Statistical Analysis

Data were assembled with Galaxy (doi:10.1093/nar/gky379) [80] and analyzed with the IBM SPSS Statistical Package v25 (IBM, Armonk, NY, USA). Linear regression analyses were performed by Pearson correlation. Differences between groups (i.e., sham, DEP- and BB-treated mice) were calculated by ANOVA with Bonferroni multiple-comparison post-hoc correction. A significance threshold of *p* ≤ 0.05 was used.

## 4. Conclusions

In summary, DEP exposure was more harmful than BB. Indeed, although both of them induce inflammatory pathways, only DEP leads to strong oxidative stress activation in lung and heart parenchyma. Data obtained indicated that the chemical composition of UFPs is the key to the differential stress response. In fact, the main contribution in oxidative stress induction after DEP instillation is associated to higher concentrations of PAHs on diesel exhausted particles as compared to BB. On the contrary, the necrotic damage caused by both kind of particles could be specifically related to their physical nature.

Here we showed that acute instillation elicited a stress response in both lung and heart characterizing a systemic damaging status. After repeated UFPs treatments, instead, BALf and lung parenchyma analysis did not disclose the presence of ongoing oxidative stress but at least a moderate cell-mediated immunity and inflammatory status. Therefore, we could hypothesize that lungs established some compensatory mechanisms in order to limit the toxic reaction and that the protective proteins, generated after a single treatment, achieved their function. Nevertheless, the inflammatory and oxidative stress pathways were activated in the cardiac tissue after repeated instillation.

Since heart histologic analysis did not show any evident deposition of UFPs, that were instead persistent in the lung, alterations in heart parenchyma could be probably due to the translocation of harmful mediators produced in the lung. Therefore, a multiorgan analysis should be interesting to investigate the hypothesis of systemic damaging effects of UFP exposure.

Our compelling evidence adds to a growing literature on the association between UFP exposure and adverse health outcomes. It is in line with studies performed on human subjects that highlighted the association of traffic-related air pollution with an acute decrement of lung function, elevated systemic inflammation [81], and the activation of several inflammatory and redox pathways (i.e., leukotriene and inflammatory mediators, cytochrome P450 etc) [82]. Furthermore, the beneficial effects of outdoor physical activity are reduced in high density traffic areas, particularly in the case of patients with chronic cardiopulmonary disease [83]. Thus, major local and international efforts need to be putted in air pollution exposure reduction, to counteract an increasingly worldwide problem for the health.

## Figures and Tables

**Table 1:** Cell count and biochemical BALf analysis. Table summarizes results of cell count and biochemical analysis in BALf of sham, BB- and DEP-treated mice after 3 h from the single intratracheal instillation (day 0) and after 24 h from the last intratracheal instillation (day 6). All the data are expressed as mean ± standard error. Concentrations under the detection limit are reported as <LOD. Statistical differences were tested by One-way ANOVA followed by Bonferroni post-hoc comparison; * *p* < 0.05 vs. sham, § *p* < 0.05 vs. BB.

	Single Instillation						Repeated Instillation					
	sham		BB		DEP		sham		BB		DEP	
	mean	st.er.	mean	st.er.	mean	st.er.	mean	st.er.	mean	st.er.	mean	st.er.
Total Cells (E+06/mL BALf)	2	0.4	1.3	0.4	3 *§	0.7	1.46	0.72	0.94	0.16	0.79	0.28
AMs%	85.7	9.8	59.5	12.2	29 *§	10	65.03	11.5	47.14	4.5	43.27	6.5
PMNs %	14	9.9	40	12.4	71 *§	10	34.56	0.39	0.43	0.25	0.35	0.16
Ls %	0.3	0.3	0.5	0.4	0.1	0.1	0.39	0.24	0.23	0.04	0.22	0.02
Total Protein (mg/mL)	0.2	0.1	0.2	0.1	0.4*§	0.1	0.21	0.02	108.91	7.7	88.32	19.7
LDH (mU/mL)	75.2	7.1	119.3 *	8.8	124.3 *	9.0	76.1	14.7	0.11	0.04	0.09	0.01
ALP (IU/L)	0.2	0.1	0.1	0.1	0.2	0.1	0.12	0.01	2.03	0.11	1.83	0.2
TAC (Trolox (nmol/mL)/Tot Pt (mg/mL))	1.7	0.1	1.9	0.2	1.5 §	0.1	2.44	0.29	6.67	1.02	5.01	0.55
ET-1 (pg/mL)	3.8	0.6	3.6	0.3	8.5 *§	1.1	6.91	1.05				
Hsp70 (ng/mL)	0.6	0.1	0.7	0.1	0.7	0.1	0.46	0.05	0.43	0.02	0.43	0.02
TNFα (pg/mL)	55.5	31.6	90.8	39.2	238.2	97	<LOD		<LOD		<LOD	
IL-6 (pg/mL)	17.1	13.3	41.1.	20.3	70.9	11.4	<LOD		<LOD		<LOD	
MMP-9 (pg/mL)	918.55	709.5	3932.9	2053.1	9100.4	3772.6	606.4	230.8	433.8	67.1	413.4	102.7

**Table 2:** Chemical compositions of UFPs from different anthropogenic sources, reported in Longhin et al., 2016. [56] Each value is expressed as mean concentrations (±SD).

Elements	Unit	DEP	BB
**Al**	ng/μg	135 ± 4	ND
**K**	ng/μg	50 ± 0.02	195 ± 12.5
**Ca**	ng/μg	198 ± 8	70 ± 4
**Fe**	ng/μg	4 ± 0.001	ND
**Zn**	ng/μg	70 ± 2	4 ± 0.001
**Cr**	ng/μg	0.04 ± 0.001	ND
**Mn**	ng/μg	0.03 ± 0.001	0.42 ± 0.03
**V**	ng/μg	0.05 ± 0.007	ND
**Ni**	ng/μg	0.02 ± 0.001	ND
**Pb**	ng/μg	0.02 ± 0.001	ND
**Total PAHs**	ng/mg	600 ± 150	50 ± 10

## Supplementary Materials

Supplementary materials can be found at https://www.mdpi.com/1422-0067/20/11/2805/s1. Table S1. All the correlations data of acute treatment. Table S2. All the correlations data of sub- acute treatment.
